# ChatGPT-4 as an Assistant for Evidence-Based Decision-Making Among General Dentists: An Observational Feasibility Study

**DOI:** 10.7759/cureus.79556

**Published:** 2025-02-24

**Authors:** Bugude Shiva Shankar, Sasankoti Mohan

**Affiliations:** 1 Periodontology, Qassim University, Buraydah, SAU; 2 Oral Medicine and Radiology, Hope Health Inc, Florence, USA

**Keywords:** ai, chatgpt, dentistry, ebdm, large language models

## Abstract

Background

Evidence-based decision-making (EBDM) is essential in contemporary dentistry. However, navigating the extensive and constantly evolving scientific literature can be challenging. Large language models (LLMs), such as ChatGPT-4, have the potential to transform EBDM by analyzing vast datasets and extracting critical information, thereby significantly reducing the time required to find evidence. This observational feasibility study investigates ChatGPT-4's potential in dental EBDM, focusing on its capabilities, strengths, and limitations.

Materials and methods

In this observational feasibility study, two independent examiners conducted interactive sessions with ChatGPT-4. Five clinical scenarios were explored using the Google Chrome web browser, accessing publicly available scientific evidence from Cochrane, ADA, and PubMed. This approach ensured compliance with the Cochrane guidelines for EBDM. Two independent dentists engaged with ChatGPT-4 in simulated real-life clinical scenarios to seek scientific information. The output from ChatGPT-4 for each scenario was assessed based on predetermined criteria. Its responses were evaluated for accuracy, relevance, efficiency, actionability, and ethical considerations using the ChatGPT-4 Response Scoring System (CRSS) and the ChatGPT-4 Generative Ability Matrix (C-GAM).

Results

ChatGPT-4 demonstrated consistent performance across all five clinical scenarios, achieving a C-GAM score of 46.4% and a CRSS score of 12 out of 28. It effectively identified relevant sources of evidence and provided concise summaries, potentially saving valuable time and enhancing access to information. No significant differences in scores were found when the responses to all clinical scenarios were analyzed independently by the two researchers. However, a notable limitation was its inability to provide specific web links directing users to relevant scientific articles. Additionally, while ChatGPT-4 offered suggestions for incorporating the latest scientific publications into decision-making, it could not generate direct links to these articles.

Conclusion

Despite its current limitations, ChatGPT-4, as a generative AI, can assist clinicians in making evidence-based decisions. It can save time compared to conventional search engines. Ethical considerations must be prioritized in training these models to ensure that clinicians make responsible, evidence-based decisions rather than relying solely on specific evidence statements provided by ChatGPT-4. This model shows its potential as an AI tool for EBDM in dentistry. Further development and training could address existing limitations and enhance its effectiveness; however, clinicians must retain ultimate responsibility for informed decisions, necessitating expertise and critical evaluation of the evidence presented.

## Introduction

Artificial intelligence (AI) represents a significant milestone in computer science, introducing both convenience and apprehension. The evolution of AI can be traced back to a 1955 research proposal paper authored by McCarthy et al. [1,2]. Copies of this seminal work are archived at Dartmouth College and Stanford University. This two-month research study proposed that with the appropriate application of technology, machines could be trained to simulate human learning and intelligence. Building upon this foundational research, the field of AI development has witnessed gradual advancements over the decades.

The scope and impact of AI extend far beyond computer science, permeating various domains, including manufacturing, search engines, healthcare, drug discovery, speech recognition, and image processing [3]. In the future of medicine, it is not unimaginable that AI could assist physicians in diagnosis and treatment planning by analyzing clinical data and radiological, biochemical, and histopathological investigations [4,5]. AI algorithms are not intended to replace human decision-making but rather to augment human capabilities, facilitating better and faster decisions [6,7]. Generative AI, a branch of AI, specializes in generating content in response to questions by leveraging pre-trained computer neural networks and algorithms [8]. In its early stages, generative AI focused on numerical data processing. However, with the rise of deep learning, its applications also expanded to encompass text, speech, and image analysis [9].

Numerous online generative AI large language model (LLM) chatbot platforms, such as ChatGPT, DALL-E, Bard, and LLaMa, have emerged. ChatGPT, an LLM chatbot developed by OpenAI, an American AI research lab, was launched on November 30, 2022. Chat Generative Pre-trained Transformer (ChatGPT) is a natural language processing model with 175 billion parameters that can generate responses to user input. ChatGPT-3.5 and ChatGPT-4 are widely used online models offered by OpenAI [10]. ChatGPT has a wide range of applications [11].

Evidence-based decision-making (EBDM) is a cornerstone of modern healthcare, ensuring that clinical decisions are grounded in the best available research evidence. However, accessing, interpreting, and applying the ever-growing volume of scientific literature can pose challenges for dental professionals. LLMs like ChatGPT offer potential solutions to these challenges. LLMs can efficiently analyze vast amounts of text data, extract key information, and provide summaries and insights, potentially aiding EBDM in dentistry. This can considerably save time and effort in accessing information. LLMs have emerged as promising tools with the potential to address the challenges associated with EBDM in healthcare.

This observational feasibility study aims to investigate the use of ChatGPT-4 for EBDM in dentistry. By exploring its capabilities, strengths, and limitations, we seek to provide valuable insights into the potential impact of ChatGPT-4 on the future of EBDM in dentistry.

In this study, we employed a structured approach to evaluate ChatGPT-4's performance in supporting dental professionals. We developed the ChatGPT-4 Generative Ability Measure (C-GAM) score to assess the AI's capabilities across five critical dimensions: accuracy in retrieving evidence-based information, relevance in finding high-quality research aligned with EBDM principles, efficiency in providing quick summaries or web links, actionability in generating clear decision statements, and ethics in recognizing professional responsibilities and limitations. This comprehensive evaluation framework allowed us to quantify ChatGPT-4's potential as an assistant for EBDM in general dentistry, providing measurable outcomes to assess its feasibility and effectiveness in clinical practice.

## Materials and methods

The following steps were implemented in sequence. This observational feasibility study was conducted over a period of approximately seven months, commencing on May 20, 2023, and concluding on December 10, 2023.

Step 1 comprised five clinical scenarios that were developed to assess EBDM, supported by a thorough electronic search to ensure the availability of relevant scientific literature in the public domain. This foundational work was crucial for validating and grounding the scenarios in current research. In Step 2, specific prompts were created to elicit essential information necessary for facilitating EBDM within each scenario, ensuring that the data gathered aligned with EBDM requirements. Moving to Step 3, a qualitative evaluation framework called the ChatGPT-4 C-GAM was established to assess ChatGPT-4's generative capabilities, encompassing five categories: accuracy, relevance, efficiency, actionability, and ethics, with assigned weightages for calculating scores for each scenario. In Step 4, a quantitative scoring metric known as the ChatGPT-4 Response Scoring System (CRSS) was developed to evaluate the quality of ChatGPT-4's responses to the prompts; this system included specific scoring criteria and point values to assess various response aspects, yielding scores that reflect overall output quality. Step 5 involved researchers conducting interactive sessions with ChatGPT-4, where they presented the developed clinical scenarios and prompts. Following this, in Step 6, ChatGPT-4's responses were meticulously recorded and scored using both the C-GAM and CRSS systems. Finally, in Step 7, a comprehensive analysis of these responses was performed based on the scores from C-GAM and CRSS, providing insights into ChatGPT-4's strengths, limitations, and potential role in EBDM in dentistry.

The materials used in this observational feasibility study are presented in Table 1.

**Table 1:** Materials used in this observational feasibility study

Materials Used
Laptop with Microsoft Windows 10 operating system
Google Chrome web browser
https://chatgpt.com website to access ChatGPT-4

In Step 1, the investigators meticulously developed five clinical scenarios to facilitate EBDM while ensuring clarity and avoiding any potential conflicts or ambiguities in the scientific information sought. Each scenario underwent independent review and refinement by the investigators to confirm their appropriateness and specificity. To verify the availability of supporting evidence, the scenarios were further evaluated by both investigators through conventional web browsing of credible professional websites, such as Cochrane Oral Health, American Dental Association (ADA), and EMBASE. This thorough pre-evaluation process allowed the investigators to gain a comprehensive understanding of the existing literature, enabling informed decisions regarding the predetermined clinical scenarios.

The investigators developed five specific, simplified clinical scenarios to evaluate ChatGPT-4's capability in facilitating EBDM. The clinical scenarios are as follows: (1) Can you help me in scientific evidence-based decision-making for this given clinical situation: In caries prevention in children, which choice should be made between topical fluoride varnish or topical fluoride gel? (2) Can you help me in scientific evidence-based decision-making for this given clinical situation: Should root canal treatment be performed in one dental visit or over several visits in permanent teeth? (3) Can you help me in scientific evidence-based decision-making for this given clinical situation: To prevent dental caries in permanent teeth of children, which is better, either pit and fissure sealant or fluoride varnish? (4) Can you help me in scientific evidence-based decision-making for this given clinical situation: For better oral hygiene in adults, which toothbrush is better, either a powered toothbrush or a manual toothbrush? (5) Can you help me in scientific evidence-based decision-making for this given clinical situation: In the management of defective resin composite dental restorations, which one is better, either total replacement of resin composite or repair of resin composite?

In Step 2, nine prompts were developed with a focus on the fundamental process of EBDM. Each clinical scenario was assessed for the quality of content generated in response to these standardized prompts. The prompts remained consistent across scenarios, with the exception of the specific clinical scenario question. This structured approach involved a sequential set of nine questions designed to ensure comprehensive and relevant information was elicited to facilitate effective EBDM.

The developed prompts are as follows: (1) I am a dental professional. Can you assist me in evidence-based decision-making for a given clinical situation? (2) Can you assist me in evidence-based decision-making for this specific clinical situation: (insert clinical scenario question)? (3) Can you search for systematic reviews published in PubMed, Cochrane Library, American Dental Association, and EMBASE? (4) Can you provide me with web links for systematic reviews published in PubMed, Cochrane Library, American Dental Association, and EMBASE? (5) Can you search for randomized controlled clinical trials (RCTs) published in PubMed, Cochrane Library, American Dental Association, and EMBASE? (6) Can you provide me with web links for RCTs published in PubMed, Cochrane Library, American Dental Association, and EMBASE? (7) Can you provide me with expert guidelines published in PubMed, Cochrane Library, American Dental Association, and EMBASE? (8) Can you provide me with web links for expert guidelines published in PubMed, Cochrane Library, American Dental Association, and EMBASE? (9) Can you provide me with a highly specific decision statement to implement for evidence-based decision-making?

In Step 3, the investigators established five broader categories and assigned weightages to evaluate ChatGPT-4's generative ability. These categories included ethics, accuracy, relevance, efficiency, and actionability, as detailed in Table 2. With the exception of ethics, the other categories were designed to assess ChatGPT-4's performance in facilitating the EBDM process while also optimizing the electronic search time for the investigators.

**Table 2:** ChatGPT-4 Generative Ability Matrix (C-GAM) EBDM: evidence-based decision-making; PICO: patient problem or population, intervention, comparison, and outcome(s)

Evaluation Category for ChatGPT-4 Generative Ability	Category Description	Weightage
Accuracy	Ability to recognize EBDM steps and implement PICO	36%
Relevance	Ability to retrieve the highest level of evidence	36%
Efficiency	Ability to save time by generating web links to scientific publications	11%
Actionability	Ability to generate EBDM statement for possible clinical application	4%
Ethics	Identify the user as a doctor and alert the user to consider updated information	7%

In Step 4, the CRSS was developed, incorporating 11 scoring criteria along with their corresponding point values to evaluate ChatGPT-4's generative abilities. These criteria were specifically designed with the five categories in mind, ensuring a comprehensive assessment of the system's performance. The details of the scoring criteria can be found in Table 3.

**Table 3:** ChatGPT-4 Response Scoring System (CRSS)

Criteria Description	Output	Score	
Is ChatGPT able to identify or acknowledge the user as a doctor?	Yes/No	Yes - 1	No - 0
Is ChatGPT able to generate specific steps to make evidence-based decisions?	Step 1: Formulate a Clear Clinical Question	Yes - 1	No - 0
	Step 2: Search for the Best Evidence	Yes - 1	No - 0
	Step 3: Appraise the Evidence	Yes - 1	No - 0
	Step 4: Apply the Evidence	Yes - 1	No - 0
	Step 5: Evaluate the Process	Yes - 1	No - 0
	Step 6: Stay Updated	Yes - 1	No - 0
Is ChatGPT able to generate PICO-based analysis for a given clinical scenario?	P (Patient Problem or Population)	Yes - 1	No - 0
	I (Intervention)	Yes - 1	No - 0
	C (Comparison)	Yes - 1	No - 0
	O (Outcome)	Yes - 1	No - 0
Is ChatGPT able to generate a response quoting systematic reviews concerning the given clinical scenario?	Yes/No	Yes - 1	No - 0
	Scientific Article Title	Yes - 1	No - 0
	Authors	Yes - 1	No - 0
	Journal	Yes - 1	No - 0
	Publication date	Yes - 1	No - 0
Is ChatGPT able to generate scientific web links for accessing systematic reviews?	Yes/No	Yes - 1	No - 0
Is ChatGPT able to generate responses quoting randomized controlled clinical trials concerning the given clinical scenario?	Yes/No	Yes - 1	No - 0
	Scientific Article Title	Yes - 1	No - 0
	Authors	Yes - 1	No - 0
	Journal	Yes - 1	No - 0
	Publication date	Yes - 1	No - 0
Is ChatGPT able to generate web links for accessing randomized controlled clinical trials?	Yes/No	Yes - 1	No - 0
Is ChatGPT able to generate responses quoting expert guidelines from professional organizations concerning the given clinical scenario?	Yes/No	Yes - 1	No - 0
Is ChatGPT able to generate web links for accessing expert guidelines from professional organizations?	Yes/No	Yes - 1	No - 0
Is ChatGPT able to give a very specific decision to implement for the given clinical scenario?	Yes/No	Yes - 1	No - 0
Is ChatGPT giving dental professionals enough suggestions to make independent professional decisions by fact-checking and analyzing the responses given?	Yes/No	Yes - 1	No - 0
Total score		28	-

In Step 5, two independent examiners conducted interactive sessions with ChatGPT-4. Each examiner was introduced separately as a dental professional seeking scientific information to address their queries. During these sessions, ChatGPT-4 was prompted to provide specific information related to a predetermined set of clinical scenarios.

The following steps were followed when interacting with ChatGPT-4: (1) Introduce the user as a dental professional seeking scientific evidence-based decision-making assistance. (2) Present ChatGPT-4 with a pre-developed clinical scenario question containing specific keywords. (3) Give the prompts to ChatGPT-4 for responses. (4) Save the responses generated by ChatGPT-4. (5) Analysis of response based on a set criteria and scoring system. (6) Analysis of ChatGPT-4 generative ability based on the following criteria: ethics, accuracy, relevance, efficiency, and actionability.

Each clinical scenario was evaluated based on the responses generated by ChatGPT-4 through a thorough and independent review by researchers. The scoring system was objectively applied to assign points for each response. The scoring criteria and allocated points are clearly outlined in the methodology section. Each clinical scenario was independently scored by two researchers after carefully reviewing the responses provided by ChatGPT-4 for the given prompts.

## Results

To assess ChatGPT-4's performance in each clinical scenario, researchers independently conducted a thorough review of its generated responses. This evaluation employed the CRSS, an objective framework assigning points based on pre-defined criteria detailed in the methodology section. Each scenario underwent independent scoring by two researchers, ensuring a comprehensive assessment. Additionally, the C-GAM was applied to qualitatively evaluate ChatGPT-4's overall generative abilities within each scenario. This combined approach provided a nuanced understanding of both the specific responses and the underlying capabilities driving them.

Presented with five distinct clinical challenges, ChatGPT-4 exhibited remarkable consistency in its performance. As shown in Table 4, all scenarios received a similar score of 12 out of a maximum of 28 on the CRSS, highlighting its overall capability across diverse domains. Independent analysis by researchers further confirmed the absence of significant score variations between scenarios.

**Table 4:** ChatGPT-4 Response Scoring System (CRSS) with scores for all five clinical scenarios CS: clinical scenario; PICO: patient problem or population, intervention, comparison, and outcome

Criteria description	Output	CS 1	CS 2	CS 3	CS 4	CS 5
Is ChatGPT able to identify or acknowledge the user as a doctor?	Yes/No	1	1	1	1	1
Is ChatGPT able to generate specific steps to make evidence-based decisions?	Step 1: Formulate a Clear Clinical Question	1	1	1	1	1
	Step 2: Search for the Best Evidence	1	1	1	1	1
	Step 3: Appraise the Evidence	1	1	1	1	1
	Step 4: Apply the Evidence	1	1	1	1	1
	Step 5: Evaluate the Process	1	1	1	1	1
	Step 6: Stay Updated	1	1	1	1	1
Is ChatGPT able to generate PICO-based analysis for a given clinical scenario?	P (Patient Problem or Population)	1	1	1	1	1
	I (Intervention)	1	1	1	1	1
	C (Comparison)	1	1	1	1	1
	O (Outcome)	1	1	1	1	1
Is ChatGPT able to generate a response quoting systematic reviews concerning the given clinical scenario?	Yes/No	0	0	0	0	0
	Scientific Article Title	0	0	0	0	0
	Authors	0	0	0	0	0
	Journal	0	0	0	0	0
	Publication date	0	0	0	0	0
Is ChatGPT able to generate scientific web links for accessing systematic reviews?	Yes/No	0	0	0	0	0
Is ChatGPT able to generate a response quoting randomized controlled clinical trials concerning the given clinical scenario?	Yes/No	0	0	0	0	0
	Scientific Article Title	0	0	0	0	0
	Authors	0	0	0	0	0
	Journal	0	0	0	0	0
	Publication date	0	0	0	0	0
Is ChatGPT able to generate web links for accessing randomized controlled clinical trials?	Yes/No	0	0	0	0	0
Is ChatGPT able to generate a response quoting expert guidelines from professional organizations concerning the given clinical scenario?	Yes/No	0	0	0	0	0
	Publication date	0	0	0	0	0
Is ChatGPT able to generate scientific web links for accessing expert guidelines from professional organizations?	Yes/No	0	0	0	0	0
Is ChatGPT able to give a very specific decision to implement for the given clinical scenario?	Yes/No	1	1	1	1	1
Is ChatGPT giving dental professionals enough suggestions to make independent professional decisions by fact-checking and analyzing the responses given?	Yes/No	1	1	1	1	1
Total score		13	13	13	13	13

This consistency is echoed in the C-GAM scores detailed in Table 5. With every scenario scoring 46.4% (13 out of 28), it is evident that ChatGPT-4's training was both thorough and effective. This consistent competency across diverse challenges suggests not only an extensive knowledge base in dental information but also the ability to effectively apply it to real-world clinical situations.

**Table 5:** ChatGPT-4 Generative Ability Matrix (C-GAM) with scores for five clinical scenarios CS: clinical scenario

Evaluation Category	Total Score Points (Sum of Maximum Criteria Scores)	CS 1	CS 2	CS 3	CS 4	CS 5
Ethics	2	2	2	2	2	2
Accuracy	10	10	10	10	10	10
Relevance	12	0	0	0	0	0
Efficiency	3	0	0	0	0	0
Actionability	1	1	1	1	1	1
Total scores (%)	28	13 (46.42%)	13 (46.42%)	13 (46.42%)	13 (46.42%)	13 (46.42%)

Furthermore, ChatGPT-4 displayed better performance in prompts related to the ethical, accurate, and actionable principles of EBDM. All scenarios achieved the highest possible scores, indicating strong alignment with these crucial aspects. However, an interesting contrast emerges when examining prompts focusing on relevance and efficiency. Notably, all scenarios received zero scores on the C-GAM in this category. This suggests a potential area for further development, where balancing ethical considerations with practical execution and resource optimization may require additional training or refinement.

Since all five clinical scenarios yielded identical CRSS and C-GAM scores, indicating a consistent level of performance from ChatGPT-4, the study opted to forego further statistical analysis. This decision was based on the assumption that additional statistical tests would not provide significant insights beyond the already observed uniformity. However, further statistical analysis might be insightful if additional research with a larger clinical scenario sample size or different evaluation criteria is undertaken in the future.

## Discussion

To the best of our knowledge, this observational feasibility study represents the first evaluation of ChatGPT-4, an LLM, as a tool for assisting dental professionals in EBDM for specific clinical scenarios. Given the nascent state of generative AI research, we developed a bespoke evaluation framework (CRSS and C-GAM) tailored to our specific research needs. We acknowledge that these criteria are open to critique and refinement by future investigators and researchers. Additionally, it is important to note that the CRSS and C-GAM scoring systems have not yet undergone formal scientific validation or testing. Our analysis revealed the following strengths and limitations of ChatGPT-4 in this context.

This observational feasibility study presents several notable strengths, particularly its pioneering exploration of AI in EBDM in dentistry. As one of the first comprehensive evaluations of ChatGPT-4's capabilities in this domain, the research paves the way for future investigations and the integration of AI tools into clinical workflows. The study also employed a systematic and structured approach, utilizing standardized clinical scenarios, structured prompts, and custom evaluation frameworks (C-GAM and CRSS) to ensure consistency and objectivity throughout the evaluation process. Ethical considerations were paramount, as the research emphasized the importance of recognizing users as dental professionals and staying updated with current information, highlighting the ethical implications of AI in healthcare. Additionally, the study focused on practical clinical scenarios that reflect real-world dental practice, enhancing the applicability of its findings. By developing two unique metrics to evaluate ChatGPT-4's generative ability and response quality, the research provided a nuanced analysis of the AI's performance in terms of accuracy, relevance, and actionability. One key finding was ChatGPT-4's potential to significantly reduce the time required for accessing and summarizing evidence compared to traditional methods, demonstrating its value in streamlining clinical workflows. The AI exhibited strong performance in delivering ethical, accurate, and actionable responses while maintaining consistency across various clinical scenarios. By identifying both the strengths and limitations of ChatGPT-4, this study sets the stage for further advancements in AI technology, contributing valuable insights to the growing field of AI in healthcare and its application in evidence-based dentistry.

This observational feasibility study presents several potential biases that could affect its findings. One concern is selection bias in the clinical scenarios, as these were crafted by the researchers, which may lead to a preference for scenarios that highlight ChatGPT-4's strengths while avoiding more challenging areas where the AI might struggle. Additionally, there is a risk of confirmation bias from the researchers, who were involved in both creating the scenarios and evaluating the results, potentially leading to an unintentional favoring of ChatGPT-4's capabilities based on how the prompts and evaluation criteria were framed. Evaluation bias is another issue, as the scoring systems (CRSS and C-GAM) developed specifically for this study have not been validated and may reflect the researchers' expectations, resulting in biased conclusions regarding the AI's performance. Technological bias is also a concern, given that ChatGPT-4 is trained on a large dataset that may contain inherent biases from its sources, potentially skewing its responses and affecting the quality of evidence provided, particularly for under-researched populations in dental care. Furthermore, the study reports uniform scores across all scenarios, suggesting a performance uniformity bias that may indicate a lack of diversity in the scenarios chosen to challenge the AI adequately. Addressing these limitations and biases in future research could lead to a more robust evaluation of ChatGPT-4's potential in clinical decision-making by incorporating a wider range of scenarios, real-world testing, and independent validation of the scoring system.

The limitations of this observational feasibility study include several critical factors that may impact the findings. One significant issue is efficiency, as criteria 5, 7, and 9 assessed ChatGPT-4's ability to generate web links to relevant scientific publications. In all five scenarios, ChatGPT-4 failed to score any points due to its inability to provide these links. While it could generate homepage links for resources such as PubMed, Cochrane Library, and the ADA, this did not significantly expedite the process of finding specific publications. The investigator would still need to employ keywords and search strategies to locate the necessary articles, highlighting a major limitation in ChatGPT-4's generative capabilities.

Additionally, the AI's failure to provide titles, authors, journals, or publication dates for systematic reviews and expert guidelines further diminished its relevance as a tool for EBDM. In our study, the clinical scenarios and prompts were carefully designed based on a review of credible, evidence-based sources such as PubMed, Cochrane, and ADA guidelines to ensure their relevance to real-world dental decision-making. While we took steps to standardize the prompts and maintain consistency across scenarios, we recognize that formal validation methods, such as expert panel review, inter-rater reliability testing, or statistical validation of examiner agreement, were not conducted. To enhance objectivity, two independent examiners evaluated ChatGPT-4's responses using predefined scoring systems (CRSS and C-GAM), ensuring structured assessment. However, we acknowledge that future studies should incorporate additional validation measures, such as piloting the prompts before full-scale testing or employing statistical tools such as Cohen's Kappa to measure inter-examiner reliability.

The study also faced limitations related to its controlled environment and small sample size of scenarios. Conducted in a simulated setting with predetermined clinical scenarios, it lacked the real-world testing necessary to address unforeseen complexities in clinical decision-making. The narrow scope of only five scenarios limits the generalizability of the findings across a broader range of clinical situations in dentistry. Moreover, the reliance on structured prompts may have constrained the AI's performance, as real-life clinical questions are often more complex and ambiguous. Compounding these issues is ChatGPT-4's limited access to updated literature due to its training data being restricted to information available until January 2022. This temporal limitation could affect its relevance in fast-evolving fields like dentistry. Lastly, the absence of statistical validation due to uniform scores across scenarios may weaken the robustness of the findings, underscoring the need for future research to address these limitations for a more comprehensive evaluation of ChatGPT-4's potential in clinical decision-making.

These limitations suggest that ChatGPT-4, in its current state, may not be suitable for independent research or decision-making tasks. Further development is needed to address these shortcomings before it can be reliably used in such contexts. However, its potential for assisting research by generating text summaries, creating outlines, or suggesting search terms should not be underestimated. With continued advancement, ChatGPT-4 could become a valuable tool for researchers, but caution and critical evaluation remain imperative at this stage.

Based on the findings from our study, we propose several recommendations to enhance the integration of AI in dental practice and improve EBDM. Firstly, future AI models should be developed with real-time access to evidence-based databases such as PubMed, Cochrane, and ADA guidelines. This integration would significantly improve the accuracy and relevance of AI-generated responses, enhancing their utility in clinical settings. Additionally, AI developers should prioritize citation transparency, ensuring that responses are well-supported by credible scientific literature, thus bolstering the reliability of AI-assisted decision-making. To enhance the evaluation of AI performance, we recommend refining clinical scenario standardization. Future studies should employ more specific and well-defined clinical cases that reflect real-world complexities, potentially utilizing expert validation processes like the Delphi method. Implementing statistical measures such as inter-rater agreement (Cohen's Kappa) would further enhance the reliability and consistency of AI performance assessment.

Our study introduced structured evaluation frameworks such as the CRSS and C-GAM. While these metrics provided a systematic approach to assess AI performance, further validation is necessary to ensure their reliability. We also recommend comparative studies between ChatGPT-4 and other AI models to determine the most suitable tools for EBDM in dentistry. Addressing ethical and practical considerations is crucial. Dental professionals should be trained to critically evaluate AI-generated responses and not rely solely on these tools for clinical decision-making. AI should be viewed as an aid rather than a replacement for human expertise. Moreover, developers should focus on improving bias detection mechanisms to ensure AI-generated recommendations are ethical, accurate, and free from misinformation. Lastly, we emphasize the need for further research, including larger sample sizes and real-world clinical testing, to better understand the practical applications of AI in dental decision-making. Longitudinal studies are essential to assess the long-term impact of AI-assisted decision-making on patient outcomes, which will help refine AI tools and establish best practices for their integration into dental practice.

The potential of LLMs for evidence-based medicine tasks has garnered increasing attention in recent years. Several studies have explored their potential applications in various areas, offering valuable insights into their capabilities and limitations. A comprehensive scoping review published in 2024, titled "Assessing the research landscape and clinical utility of large language models: a scoping review," investigated the existing research on LLMs in evidence-based medicine across diverse clinical fields. Park et al. recommended an optimistic yet cautious approach to integrating LLMs into clinical settings, emphasizing the need for further development to address challenges before widespread adoption [12]. They highlighted that while conversational prowess and medical knowledge make LLMs like ChatGPT and Bard intriguing for clinical applications, their path to adoption is fraught with hurdles. Issues such as hallucinations, lack of transparency, and inconsistent outputs raise concerns about accuracy and patient safety. Ethically and legally, issues like patient consent, legal liability, and data privacy add another layer of complexity [12].

Tang et al. critically examined the strengths and weaknesses of LLMs like GPT-3.5 and ChatGPT in summarizing medical evidence across diverse clinical fields. They used both automated and human evaluations to assess summary quality, revealing a disconnect between automatic metrics and human judgment. Notably, they identified a range of error types specific to medical evidence summarization, highlighting the potential for LLMs to generate factually inaccurate, misleading, or uncertain summaries. Additionally, the study found that LLMs struggle to identify core information and become more prone to errors when summarizing longer texts [13].

Van Veen et al. tested an LLM for automatically summarizing research articles, finding it comparable to human summaries in brevity, accuracy, and key information retrieval. This suggests LLMs could significantly streamline systematic reviews by quickly screening articles, extracting findings, and potentially aiding in relevancy assessment. However, human review remains crucial to ensure completeness and capture nuanced details. Overall, LLMs show promising potential to revolutionize research synthesis in healthcare, demanding further research to optimize their impact on systematic review quality [14].

Qureshi et al.'s investigation into ChatGPT's potential for systematic reviews reveals promising glimpses but ultimately concludes the technology remains insufficiently developed for such applications. While researchers report some helpful ChatGPT responses to SR-related prompts, they emphasize the tool's infancy and caution against its uncritical use by non-experts. Deceptive validity masks inaccuracies in much of the output, necessitating meticulous verification by human reviewers [15]. While various researchers have expressed their views on ChatGPT's ability in scientific writing and its pros and cons [16-18], no prior research has specifically examined ChatGPT-4's capability in facilitating EBDM for clinical scenarios.

## Conclusions

Our observational feasibility study highlights the potential of ChatGPT-4 for EBDM in dentistry. By leveraging its capabilities for information retrieval, knowledge synthesis, and decision support, ChatGPT-4 as an LLM can potentially streamline EBM workflows and improve clinical decision-making. ChatGPT-4 shows promise in supporting some aspects of EBDM for dental professionals. Its strengths in ethical awareness, EBDM process adherence, and information retrieval guidance demonstrate potential for valuable contributions. However, its limitations in efficiency and information provision about specific publications significantly hinder its ability to streamline the EBDM process.

Our research on ChatGPT-4's specific capabilities and limitations contributes valuable insights to this ongoing discussion, paving the way for the development of more powerful and reliable LLM-based tools for dental professionals' EBDM workflows. Ethical considerations must be carefully considered during the training of such models to ensure that clinicians make their own informed, evidence-based decisions rather than relying solely on the specific evidence statements provided by ChatGPT-4. Further training of ChatGPT could address its current limitations and unlock its full potential. Ultimately, the responsibility for making the final decision rests with the clinician.
